# Long-Term Follow-Up of a Complicated Rupture of Multiorgan Hydatid Cysts: A Case Report

**DOI:** 10.7759/cureus.87647

**Published:** 2025-07-10

**Authors:** Ahmed Taha

**Affiliations:** 1 General Surgery, Bursa Yüksek İhtisas Training and Research Hospital, Bursa, TUR

**Keywords:** acute surgical abdomen, hydatid cyst, liver, rupture, spleen, stomach, surgery

## Abstract

Hydatid disease (echinococcosis) remains a serious health problem in underdeveloped countries. Although the liver is the primary site of infection, other organs may also be involved. Multifocal involvement of multiple abdominal organs is rare but can occur. Spontaneous cyst rupture constitutes a critical surgical emergency with significant mortality. We discussed a case managed surgically for perforated hydatid disease. A two-year follow-up of a giant complicated cyst involving the liver, stomach, and spleen was presented.

## Introduction

Hydatid disease (echinococcosis) remains a serious global health concern, primarily affecting regions with widespread animal husbandry and agriculture [1]. Worldwide incidence ranges from 1 to 150 per 100,000 individuals. Echinococcus granulosus is the principal causative agent, with hepatic involvement most common (60%-70%), followed by pulmonary (20%) and splenic (0.5%-4%) involvement; other organ manifestations are rare [1,2]. Combined antiparasitic and surgical therapy constitutes standard management. Cysts may rarely perforate spontaneously or post-trauma, causing a life-threatening acute abdomen [3]. Dissemination of cyst contents can trigger severe anaphylaxis, underscoring the imperative for urgent intervention and strict adherence to combined therapies to reduce morbidity/mortality [4]. Calcified/inactive cysts (Gharbi type IV) are managed medically, rarely requiring surgery. For types I-III cysts, open or laparoscopic techniques are tailored to disease stage [5]. In perforation cases, immediate surgery is critical to prevent anaphylaxis and minimize peritoneal contamination [6]. We present a high-risk case of long-standing complicated hydatid disease involving nearly the entire left hepatic lobe with extension to the gastric serosa and spleen, requiring emergency laparotomy for perforation.

## Case presentation

**Figure 1:**
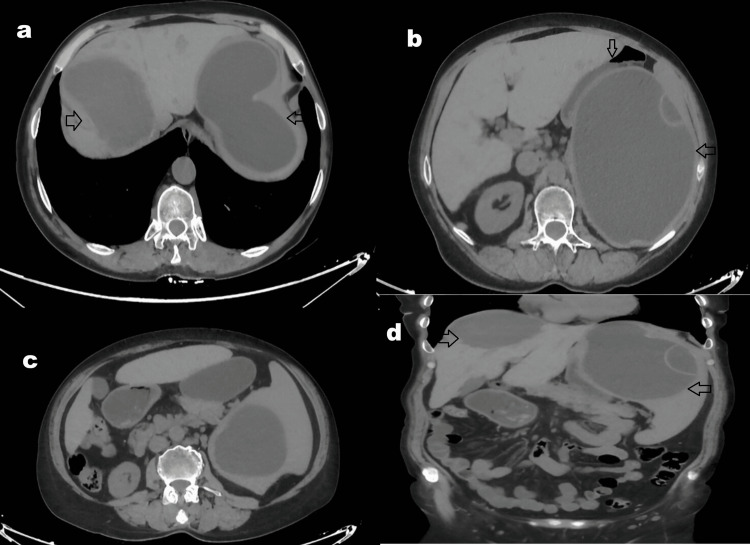
CT images obtained during the conservative follow-up period, approximately three months before surgery. Changes will be added. CT images taken three months before surgery show an isolated large cyst in the right hepatic lobe (a). The cyst in the left hepatic lobe involves both the anterior wall of the stomach (b) and a large portion of the spleen (c, d). The cysts are not fully calcified. Cyst in the right hepatic lobe (arrow >); cyst extending from the left hepatic lobe to the stomach and spleen (arrow <); stomach (arrow ∨).

A 62-year-old female with no significant comorbidities or prior surgical history had been under long-term surveillance at an external tertiary center for a complicated hydatid cyst deemed high risk for surgical intervention. Current management included oral antiparasitic therapy (albendazole: 2 x 200 mg/day). Imaging records, including an abdominal ultrasound performed three months prior, revealed a heterogeneous semisolid lesion (8 × 7 cm) in the posterior segment of the right hepatic lobe, consistent with a type IV hydatid cyst. Splenic contour, size, and parenchymal echogenicity appeared normal; however, a large cystic structure (140 × 133 mm) was identified at the upper pole of the spleen, containing dense internal content suggestive of daughter vesicle cysts. Contrast-enhanced computed tomography (CT) findings corroborated these observations (Figure 1).

The patient presented acutely to the emergency department in June 2023 with sudden-onset severe abdominal pain, nausea, vomiting, and dizziness, necessitating urgent evaluation for suspected cyst rupture or complications.

On examination, the patient was conscious, alert, and cooperative. Abdominal assessment revealed significant distension with diffuse tenderness, rebound tenderness, and involuntary guarding in all quadrants - findings consistent with peritonitis.

Diagnostic assessment: heart rate, 103/minute; blood pressure, 90/65 mmHg; temperature, 36.9 °C; leukocyte count (WBC), 20.32 × 10³/mm³; neutrophils, 87.8%; hemoglobin (HGB), 9.3 g/dL; platelets (PLT), 248 × 10³/mm³; C-reactive protein (CRP), 0.4 mg/L (Table 1).

**Table 1:** Two-year laboratory values. WBC, white blood cell count; CRP, C-reactive protein; IHA, indirect hemagglutination assay (for hydatid cyst).

	WBC (x10³/mm³)	Neutrophil (%)	CRP (mg/L)	Hemoglobin (g/dL)	Platelets (x10³/mm³)	Sedimentation (mm/h)	IHA titration
Preoperative (six months)	6.49	59.9	47.7	10.7	239	44	1:160
Operation day	20.32	87.8	249.7	9.3	248	-	-
5th DAY	17.48	84.1	159	9	428	72	-
2nd week	10.10	46.3	8.7	9.4	1277	46	1:10240
3rd month	7.84	31.9	5.19	10.3	474	11	1:1280
6th month	9.49	50.6	3.71	11.3	492	14	1:1280
1st year	8.40	41.8	2.9	11	419	10	1:640
2nd year	5.19	51.6	4.1	12	434	15	1:160

**Figure 2:**
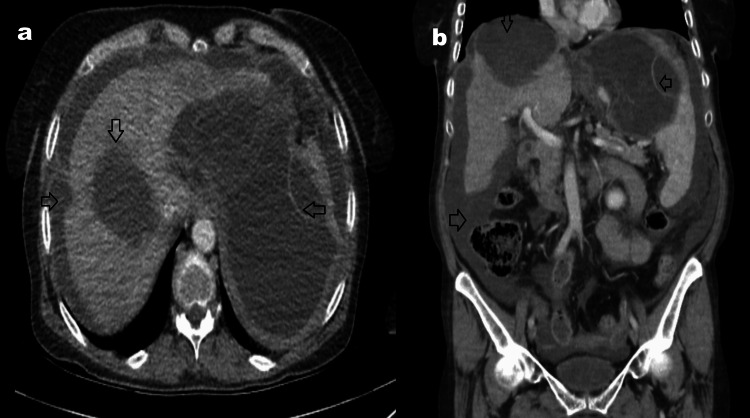
CT images taken in the emergency room. CT images obtained upon the patient's admission to the emergency department showed that the cyst wall in the right hepatic lobe (a, b) remained relatively intact, while the cyst in the left lobe had decreased in size. There was also widespread free fluid in the abdominal cavity. It was noted that the free fluid had the same density (b) as the cyst contents. Cyst in the right hepatic lobe (arrow ∨); volume loss and perforation findings in the cyst on the left (arrow <); free fluid in the abdomen (arrow >).

Upon admission, a Focused Assessment with Sonography in Trauma (FAST) exam demonstrated significant free fluid in all abdominal quadrants, suggestive of hemoperitoneum or cyst rupture. An emergency CT scan revealed equivocal findings concerning perforation, although definitive confirmation was limited due to imaging artifacts (Figure 2). Given the high clinical suspicion of an acute intra-abdominal catastrophe, prompt surgical exploration was deemed imperative. A diagnosis of ruptured hydatid cyst presenting with acute abdomen was confirmed.

After preoperative preparation, a wide subcostal incision was made to access the abdominal cavity. Exploration revealed copious free fluid, which was aspirated, followed by systematic organ examination. A consolidated cystic mass was identified with extensive involvement of hepatic segments II and III, the anterior gastric wall, and spleen, occupying the left upper quadrant with a medial perforation. An 8-10 cm well-circumscribed cystic lesion in the right hepatic lobe showed no perforation evidence. Given splenic hilar involvement, resection began at the hepatic site: segments II and III, with the adherent cyst, were excised en bloc. Hemostasis was achieved via sequential ligation and electrocautery.

A gastric wedge resection of the anterior wall was performed using a linear stapler, reinforced with interrupted Lembert sutures to ensure serosal integrity. Subsequent splenic dissection revealed complete organ encasement, necessitating total splenectomy en bloc with the cyst. Finally, cholecystectomy and complete right hepatic cyst excision were completed. The abdomen was copiously irrigated with saline, and four closed-suction drains were placed in the dependent quadrants. The six-hour procedure was complicated by persistent hypotension, prompting Intensive Care Unit (ICU) transfer for hemodynamic stabilization.

**Figure 3:**
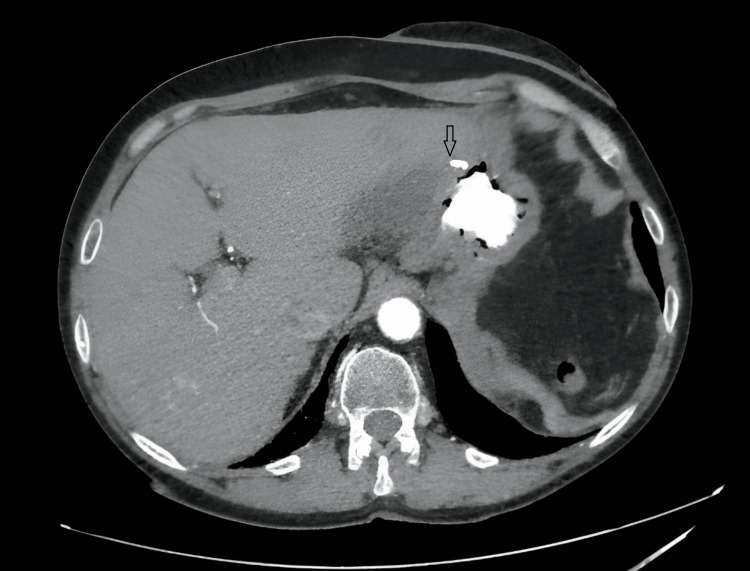
CT image obtained on the 5th postoperative day. In the early postoperative CT images, the staple line (arrow) in the anterior stomach is visible. No contrast material leakage, free fluid, or infectious content is observed.

The patient was extubated on postoperative day 2 (POD 2). By POD 5, CT confirmed satisfactory resolution of intra-abdominal pathology (Figure 3), and partial hematologic recovery (Table 1) enabled ward transfer. Drains were removed after two weeks of observation, and the patient was discharged with outpatient follow-up instructions.

**Figure 4:**
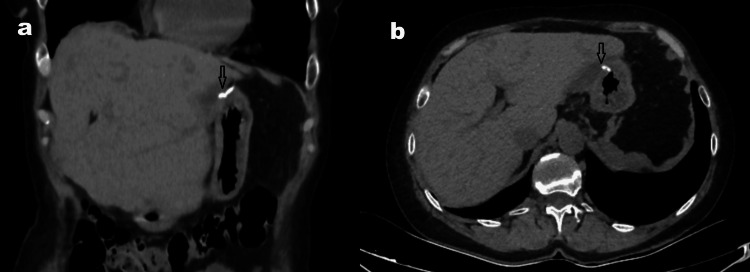
CT images obtained two years after surgery. CT images taken after a long follow-up period show decreased liver volume (a), the staple line in the anterior stomach (arrow), and an empty splenic lobe (a, b). No recurrence is observed.

Over a 24-month surveillance period (Table 1, Figure 4), no postoperative complications, including recurrence or secondary infection, were observed. Albendazole treatment was continued at a dose of 2 x 400 mg/day for the first year after surgery. After the indirect hemagglutination assay (IHA) titer decreased to 1:640, the dose was reduced to 2 × 200 mg/day (Table 1).

## Discussion

The economic burden of maintaining hygienic conditions, particularly in open or pastoral environments, remains prohibitive in resource-limited regions. These challenges are compounded by inadequate public health infrastructure and limited access to preventive measures [7]. Consequently, hydatid cyst disease persists as a major public health challenge in underdeveloped nations, perpetuated by zoonotic transmission cycles involving livestock and deficient sanitation practices [1,4]. Hydatid cysts most frequently manifest as solitary or multifocal lesions within the hepatic parenchyma, accounting for 50%-70% of cases. Less commonly, involvement of the lungs (10%-30%) or spleen (2%-6%) is observed. Multiorgan dissemination occurs in approximately 5%-10% of cases, reflecting systemic parasitic dissemination. Notably, luminal organ involvement (e.g., gastrointestinal or urinary tracts) is atypical, as Echinococcus granulosus larvae preferentially localize to solid abdominal and thoracic viscera. This anatomical predilection correlates with the parasite’s hematogenous dissemination patterns and host tissue tropism [1,2,8]. Diagnosis of hydatid cysts is typically established via imaging modalities, including ultrasonography (USG), CT, and magnetic resonance imaging (MRI) [8,9]. Serological agglutination tests may supplement diagnostic evaluation by assessing parasitic activity and immunological response [9]. Surgical intervention remains the primary treatment for active cysts, particularly in symptomatic or high-risk cases. Conservative management through surveillance is reserved for nonactive, calcified type IV cysts, as defined by many studies in the literature, which exhibit minimal risk of complications [10]. In types I-III (active or transitional phases), hydatid cysts are associated with pathognomonic complications, including abscess formation, intraperitoneal rupture (risk of peritoneal dissemination or anaphylaxis), biliary rupture (leading to obstructive jaundice or recurrent cholangitis), and hemobilia secondary to vascular erosion [11]. While spontaneous cyst rupture occurs more frequently in pulmonary hydatidosis, hepatic cysts, particularly those exceeding 10 cm in diameter, carry a rupture risk of 1%-8%. This risk escalates to approximately 30% in cases complicated by abdominal trauma [12]. Hydatid cyst perforation represents a severe and potentially fatal complication, with reported mortality rates of 5%-10%. Delayed intervention may precipitate life-threatening sequelae, including anaphylactic shock, secondary infections (e.g., bacterial peritonitis), and intraperitoneal dissemination of protoscolices [4,6,11,12]. Radiological diagnosis of rupture relies on imaging findings such as the *water lily sign* (collapsed endocyst membrane) on USG or CT, though this pathognomonic feature may not manifest in early-stage ruptures [9,13]. The presence of free intraperitoneal fluid, occasionally accompanied by pneumoperitoneum, is a hallmark radiological finding in hydatid cyst rupture. Clinically, this manifests as an acute abdomen, characterized by peritoneal signs and systemic instability. Surgical intervention remains the definitive treatment for confirmed or suspected rupture, as conservative measures fail to address peritoneal contamination or anaphylactic risk [4,6,9]. Emergent laparotomy with cyst excision, peritoneal lavage, and source control is critical to mitigate life-threatening complications such as disseminated echinococcosis or septic shock [4,6]. The introduction of robotic surgery, with recent developments, significantly reduces mortality rates, especially when timely intervention is performed before the onset of irreversible hemodynamic deterioration. [3,14].

The presented case exemplifies the elevated morbidity associated with large, multiorgan hydatid cysts characterized by complex anatomical involvement. The patient’s prolonged intraoperative course and significant mortality risk, attributable to peritoneal dissemination and systemic inflammatory response, underscore the challenges inherent in managing advanced-stage echinococcosis. Despite these adversities, successful recovery was achieved through meticulous surgical intervention. This outcome reinforces the critical importance of prioritizing elective surgical management for high-risk cysts, even in anatomically complex scenarios. While perioperative risks remain substantial, they are demonstrably lower than the catastrophic mortality associated with spontaneous rupture, which often increases in cases of delayed presentation. Thus, early intervention, guided by multidisciplinary risk stratification, may optimize outcomes in similarly complex presentations.

## Conclusions

Hydatid cyst multifocal presentations, involving concurrent visceral and/or various systems, are observed in rare cases. Spontaneous cyst rupture constitutes a critical abdominal emergency, often resulting in peritoneal dissemination of protocoleces, anaphylactic shock, or secondary bacterial peritonitis, with high mortality rates. Given the high morbidity and mortality associated with rupture, appropriate surgical intervention for large (>10 cm) or anatomically complex cysts, even in asymptomatic patients, is generally advocated. Prophylactic resection before rupture may mitigate catastrophic outcomes, as elective procedures are associated with significantly lower mortality compared to emergency surgeries following rupture. Thus, early multidisciplinary risk assessment and adherence to surgical guidelines are imperative to optimize patient outcomes in advanced-stage hydatidosis.
